# Intercellular Adhesion Molecule-1 Gene Polymorphisms and Susceptibility to Cervical Cancer in the Northern Chinese Han Population

**DOI:** 10.3389/fgene.2021.668539

**Published:** 2021-07-27

**Authors:** Yanan Feng, Xiaoying Li, Qi Ma, Shuang Zhang, Manning Zhu, Songxue Li, Lei Fang, Jiawei Tian, Litao Sun

**Affiliations:** ^1^Department of Ultrasound, The 2nd Affiliated Hospital of Harbin Medical University, Harbin, China; ^2^Department of Ultrasound, Harbin Red Cross Central Hospital, Harbin, China; ^3^Department of Ultrasound, People’s Hospital of Zhejiang Province, Hangzhou, China

**Keywords:** cervical cancer, ICAM-1, SNPs, genetic association, multiplex polymerase chain reaction

## Abstract

Many epidemiological studies have confirmed that *ICAM-1* gene single-nucleotide polymorphisms (SNPs) are associated with susceptibility of various cancers, but there are relatively few studies on the relationship between *ICAM-1* gene polymorphisms and the risk of cervical cancer. Therefore, we aimed to explore the potential role of *ICAM-1* gene polymorphisms and the combined effect of SNPs in the pathogenesis of cervical cancer in Han women in northern China. This case–control group includes 488 cases of cervical cancer, 684 cases of cervical precancerous lesions, and 510 healthy females. Multiplex polymerase chain reaction (PCR) combined with the next-generation sequencing method was used for the determination of gene polymorphisms (rs5498, rs3093030, and rs281432). In our study, we divide cervical cancer into two subgroups: cervical squamous cell carcinoma (CSCC) group and cervical adenocarcinoma (CAC) group. We analyzed the alleles and genotypes of all research subjects using multivariate logistic regression analysis combined with 10,000 permutation tests. In addition, we also analyzed the distribution of haplotypes of the three SNPs in cervical cancer and cervical precancerous lesions. We found that the T allele and the dominant model of rs3093030 were associated with the susceptibility of cervical cancer (*p* = 0.042, *p* = 0.040, respectively). However, the significance disappeared after the Bonferroni correction for multiple testing (*p* > 0.05). For rs5498, its mutant gene G, the codominant model, and the dominant model could reduce the risk of CAC (*p* = 0.009, *p* = 0.028, *p* = 0.011, respectively). Significant differences remained after Bonferroni correction (*p* < 0.05, all). In addition, the frequency of haplotype “CTG” was significantly lower in the CAC group than in the controls. In conclusion, the study suggested that *ICAM-1* gene polymorphisms may have a potential role in the pathogenesis of cervical cancer in the northern Chinese Han population.

## Introduction

Cervical cancer, a serious public health concern, is the fourth most commonly diagnosed cancer and also the fourth leading cause of cancer deaths in women worldwide (Torre et al., 2015). Eighty-three percent of these deaths are reported in developing countries (Berumen-Campos, 2006). Despite that cervical cytology screening has been common in recent decades, cervical cancer is still one of the most common cancers in women (Chen et al., 2016c; Zheng et al., 2016; Siegel et al., 2017). Early cervical cancer can be treated by surgery or radiotherapy and chemotherapy with good outcomes, but metastatic cervical cancer is still incurable (Uyar and Rader, 2014). Therefore, it is particularly important to find an effective diagnosis method in the early stage. Since single-nucleotide polymorphisms (SNPs) are important genetic markers for the localization of complex diseases such as human cancer (Shastry, 2009; Tan, 2017), the study of certain SNPs related to cervical cancer will contribute to the early diagnosis and treatment of cervical cancer.

Intercellular adhesion molecule-1 (ICAM-1), also known as CD54, is a member of the immunoglobulin superfamily (IGSF) of adhesion molecules and plays an important role in mediating adhesion reactions. The *ICAM-1* gene is located on chromosome 19p13.2 and has five extracellular immunoglobulin-like domains, a single transmembrane, and a short cytoplasmic tail (Springer, 1990; Staunton et al., 1990). There are two forms of ICAM-1 *in vivo*, namely, membrane ICAM-1 (mICAM-1) and soluble ICAM-1 (sICAM-1). ICAM-1 exists in the extracellular form of sICAM-1, which is mainly produced by mICAM-1 entering the blood after lysis by protease. ICAM-1 plays an important role in promoting adhesion in the inflammation site, controlling tumor progression and metastasis, and regulating the body’s immune response, and is widely distributed in a variety of cell types, including fibroblasts, keratinocytes, vascular endothelial cells, macrophages, and glandular epithelial cells and stromal cells of the uterus (Dustin et al., 1986; Larson and Springer, 1990; Tabibzadeh and Poubouridis, 1990).

Interestingly, several studies have shown that *ICAM-1* gene polymorphisms were associated with susceptibility to breast cancer (Kammerer et al., 2004), gastric cancer (Tian et al., 2012), non-small cell lung cancer (Thanopoulou et al., 2012), hepatocellular carcinoma (Chen et al., 2016b), prostate cancer (Chen et al., 2006), melanoma (Natali et al., 1990), and ovarian cancer (Gardner et al., 1995), suggesting that the SNPs of the *ICAM-1* gene may be common genetic factors that affect individual cancer susceptibility. To prove this hypothesis, we conducted a replication study to assess the relationship between *ICAM-1* gene polymorphisms and the risk of cervical cancer in the northern Chinese Han population, which was different from the population studied by Sun et al. (2016).

## Materials and Methods

### Participants

A total of 488 cervical cancer patients, 684 cervical precancerous lesion patients, and 510 healthy females were recruited from the second affiliated hospital of Harbin Medical University in Heilongjiang Province, China, from September 2014 to October 2018 in this study. The cervical cancer group consists of 395 cases of cervical squamous cell carcinoma (CSCC), 34 cases of cervical adenocarcinoma (CAC), and 59 cases of other pathological types of cancer. On this basis, we have further analyzed the relationship between common pathological types of cervical cancer, CSCC and CAC, and *ICAM-1* gene polymorphisms. In addition, for cervical precancerous lesions, only patients with high-grade intraepithelial lesions, which included moderate and severe dysplasia and carcinoma *in situ*, were recruited. All the patients obtained their tissue specimens by surgery or biopsy and were further diagnosed by the pathologists of the Second Affiliated Hospital of Harbin Medical University. At the same time, the exclusion criteria were as follows: cervical benign lesions, cervical benign tumors, other cervical malignant tumors, and patients with cervical lesions who have undergone preoperative radiotherapy and chemotherapy. These healthy women were selected from a population of TCT-negative, cancer-free, or cancer-free family history who had undergone regular physical examinations at the Second Affiliated Hospital of Harbin Medical University or who volunteered to participate in epidemiological investigations. The cases and controls were non-probabilistic continuous samples. All participants were biologically unrelated Han Chinese female living in northern China.

The research was approved by the ethics committee of the Second Affiliated Hospital of Harbin Medical University, and informed consent for participation in this study was obtained from all subjects.

### Selection of SNPs

Based on Sun et al.’s (2016) study of cervical carcinogenesis in Taiwanese women, as well as published candidate gene-association studies, and combined with the characteristics of the East Asian population in HapMap, we selected three *ICAM-1* gene SNPs, rs5498, rs3093030, and rs281432. The minor allele frequency of the three SNPs is shown as follows: rs5498 P_*G*_ = 0.381, rs3093030 P_*T*_ = 0.317, and rs281432 P_*G*_ = 0.360. Of the SNPs, rs5498 (A > G) was located in the exon region, rs3093030 (C > T) was SNP between the *ICAM-1* and *ICAM-4* genes, and rs281432 (C > G) was located in the intron region.

### Extraction of DNA

Peripheral venous blood was collected from all subjects on admission and placed in a 2% EDTA-Na_2_ anticoagulant tube, which was then refrigerated at −80°C until DNA extraction. The genomic DNA of all subjects was extracted in strict accordance with the standard steps of the TIANamp Genomic DNA Kit (Tiangen Biotech, Beijing, China). DNA samples were amplified by PCR. All genotyping experiments were completed by Shanghai Biowing Applied Biotechnology Company^1^ using the multiplex PCR method combined with next-generation sequencing methods (Chen et al., 2016a). Primer 3 online software (Version 0.4.0)^2^ was used to design amplification primers for rs5498, rs3093030, and rs281432 of the *ICAM-1* gene. The amplification primers are as follows: for rs5498 sense primer 5′-TGCCCATCGGGGAATCAG-3′ and anti-sense primer 5′-CAGTGATGATGACAATCTCATACC-3′, for rs3093030 sense primer 5′-TAATAAAGCTTTCTCAACTGCCTC-3′ and anti-sense primer 5′-TCTGGAGAGGAGTCTTTAGTTTTC-3′, and for rs281432 sense primer 5′-GATTGATGGGAGGAAGGGTG-3′ and anti-sense primer 5′-TGTCCTTCCCTTCTTGAATACG-3′. After PCR amplification, the PCR products were purified by TIANgel Midi Purification Kit (Tiangen Biotech, China). The purified PCR products were then paired-end sequenced (2 × 150 bp) by Illumina HiSeq X Ten platform according to the SOP. The readings were aligned to the human reference genome using Burrows–Wheeler Aligner (BWA, v0.7.12) (Li, 2011), and Samtools (v0.1.19) was used for SNP calling and genotyping.

### Allele, Genotype, and Haplotype Association Analysis

In order to find the relationship between *ICAM-1* gene polymorphisms and cervical cancer and cervical precancerous lesion susceptibility, we used the following models: codominant, dominant, and recessive models. Allele frequencies and genotype distribution (under 2-df codominant, 1-df dominant, and 1-df recessive models) were compared between groups using the χ^2^ test of independence with a 2 × 2 contingency. Haplotype analysis between controls and cases was implemented using the SHEsis software^3^ (Shi and He, 2005; Li et al., 2009). Where appropriate, the odds ratios (OR) with corresponding 95% confidence intervals (CI) were calculated. Empirical *p* values were calculated by 10,000 permutation tests using the Max(T) permutation procedure implemented in PLINK 1.9 software^4^ (Purcell et al., 2007). Moreover, multiple comparisons were counteracted by using the Bonferroni correction in PLINK 1.9 software. *P* < 0.05 was considered statistically significant.

### Multivariable Logistic Regression Analysis

Multivariable logistic regression analysis (using SPSS V21.0 for Windows) was used to assess the association of the allele and genotype of each SNP with cervical cancer and cervical precancerous lesion after adjustment for age.

## Results

### SNP Genotype and Quality

Linkage disequilibrium among the SNPs was tested using SHEsis software. All SNPs were found to be in Hardy–Weinberg equilibrium in the case group and controls (Table 1). In order to ensure the accuracy of the study, we set the quality control for 73 samples with an accuracy rate of 98.5%, providing evidence for the reliability of subsequent studies.

**TABLE 1:** Hardy–Weinberg equilibrium test results of *ICAM-1* SNPs in the cervical cancer and control groups.

SNPs	Control		CC case	
		
	χ^2^	*p*	χ^2^	*p*
rs281432	0.011	0.915	0.668	0.796
rs3093030	0.043	0.835	0.189	0.664
rs5498	0.028	0.866	0.899	0.343

*CC, cervical cancer.*

### Clinical Characteristics of the Study Population

The characteristics of the individuals enrolled in our study are listed in Table 2. The ages of the subjects with cervical precancerous lesions and cervical cancer and the controls were significantly different (*p* < 0.05). In the cervical cancer group, there were 395 patients with squamous cell carcinoma, 34 patients with adenocarcinoma, and 59 patients with other pathological types.

**TABLE 2:** The characteristics of all subjects in this study.

Demographic clinicopathologic data	CC case (*n* = 488)	CPL case (*n* = 684)	Controls (*n* = 510)
**Age**			
Median (range)	49 (20–72)*	41 (21–72)*	39 (25–68)
**Menarche age**			
<15 years	229	387	
≥15 years	243	285	
Missing data	16	12	
**Amenorrhea**			
Yes	227	127	
No	248	542	
Missing data	13	15	
**HPV**			
(+)	90	387	
**SCC**			
≤1.5	194	459	
>1.5	242	61	
Missing data	52	164	
**Histology**			
Squamous	395		
Adenocarcinoma	34		
Other subtypes	59		

CC, cervical cancer; CPL, cervical precancerous lesions.

*Compared with the controls, there was a statistically significant difference.

### *ICAM-1* SNPs and Cervical Cancer

We genotyped three *ICAM-1* gene polymorphisms as follows: rs5498, rs3093030, and rs281432. The genotype and allele frequencies of each SNP between the controls and cervical cancer patients and subgroups, CSCC and CAC, are summarized in Table 3. The results showed that the frequency of the rs3093030 C/T allele was significantly different between the controls and cervical cancer patients (*p* = 0.042). However, logistic regression analysis-adjusted age factor and Bonferroni correction showed no significant statistical significance (*p* = 0.060, *p* = 0.125, respectively). The frequency of the rs5498 A/G allele was significantly different between the controls and CAC patients (*p* = 0.009). The results of *p* after logistic regression analysis-adjusted age factors showed that the frequency of the rs5498 A/G allele in CAC patients was also significantly different from controls (*p* = 0.016). This difference remained statistically significant after Bonferroni correction (*p* = 0.028). For the rs281432 C/G allele, compared with the controls, there was no significant correlation in either the cervical cancer patients or the subgroups, CSCC and CAC (*p* > 0.05).

**TABLE 3:** Distribution of genotypes and alleles of three SNPs in the cervical cancer and control groups.

	Controls (n = 510)	CC group (n = 488)		*P*	P*	*P* ^#^	CSCC group (n = 395)		*P1*	P1*	*P1* ^#^	CAC group (n = 34)		*P2*	P2*	*P2* ^#^
**rs5498**																
AA	239	249	Codominant	0.432	0.297	0.685	195	Codominant	0.729	0.518	0.959	24	Codominant	**0.028**	**0.041**	**0.029**
AG	218	191	Dominant	0.207	0.164	0.425	159	Dominant	0.472	0.399	0.749	9	Dominant	**0.011**	**0.012**	**0.027**
GG	48	45	Recessive	0.903	0.812	0.987	38	Recessive	0.924	0.626	0.909	1	Recessive	0.226	0.302	0.509
A/G	0.689/0.311	0.710/0.290	Allele	0.304	0.330		0.700/0.300	Allele	0.611	0.664		0.838/0.162	Allele	**0.009**	**0.016**	
**rs3093030**																
CC	264	284	Codominant	0.114	0.148	0.203	228	Codominant	0.189	0.265	0.452	22	Codominant	0.301	0.287	0.269
CT	205	172	Dominant	**0.040**	0.052	0.189	139	Dominant	0.069	0.103	0.402	11	Dominant	0.157	0.122	0.280
TT	38	29	Recessive	0.343	0.428	0.792	25	Recessive	0.516	0.612	0.956	1	Recessive	0.339	0.450	0.740
C/T	0.723/0.277	0.763/0.237	Allele	**0.042**	0.06		0.759/0.241	Allele	0.085	0.130		0.809/0.191	Allele	0.123	0.125	
**rs281432**																
CC	235	222	Codominant	0.948	0.897	0.999	180	Codominant	0.942	0.919	0.984	18	Codominant	0.675	0.659	0.653
CG	217	201	Dominant	0.819	0.962	0.997	160	Dominant	0.748	0.838	0.992	14	Dominant	0.496	0.408	0.718
GG	49	43	Recessive	0.770	0.669	0.954	35	Recessive	0.824	0.687	0.961	2	Recessive	0.459	0.539	0.849
C/G	0.686/0.314	0.692/0.308	Allele	0.760	0.878		0.693/0.307	Allele	0.730	0.738		0.735/0.265	Allele	0.392	0.369	

*CC, cervical cancer; CSCC, cervical squamous cell carcinomas; CAC, cervical adenocarcinoma.*

*P*: controls vs. CC; *P1*: controls vs. CSCC; *P2*: controls vs. CAC.

*Age was adjusted.

*^#^The *p* value was calculated using 10,000 permutations for each model to correct the multiple tests.*

*Codominant with degree of freedom 2, dominant and recessive models with degree of freedom 1. *p* values < 0.05 are shown in bold.*

For the rs5498 SNP, the codominant model and dominant model distributions were significantly different in controls and CAC patients (*p* = 0.028, *p* = 0.011, respectively). Moreover, after performing 10,000 permutations, the *p* value was marginally significant (codominant model *p* = 0.029, dominant model *p* = 0.027). After Bonferroni correction, we found that there were marginal differences under the codominant model and dominant model (*p* = 0.049, *p* = 0.042, respectively). For the rs3093030 SNP, compared with the controls, dominant model distribution in cervical cancer patients was significantly different (*p* = 0.040). After 10,000 permutations, the *p* value of the dominant model was 0.189, and the significance disappeared after Bonferroni correction for multiple testing, indicating that the controls have no significant association with cervical cancer patients. Neither the genotype nor the allele frequencies of the rs281432 SNP were significantly different compared controls with cervical cancer patients or subgroups, CSCC and CAC.

### *ICAM-1* SNPs and Cervical Precancerous Lesions

Table 4 shows the distribution of the genotype and allele frequencies of the three SNPs of the *ICAM-1* gene in the healthy control group and the cervical precancerous lesion group. With healthy women as a control, there was no significant difference between the three SNPs and the genetic susceptibility of patients with cervical precancerous lesions (*p* > 0.05, all). We further analyzed the relationship between cervical precancerous lesions and cervical cancer (Table 4); neither the genotype nor the allele frequencies of the polymorphisms were significantly different (*p* > 0.05, all). After using logistic regression analysis to adjust for the age factors, there was no difference with cervical precancerous lesions (*p* > 0.05, all). There was no statistical significance in the results obtained by 10,000 permutations. Similarly, the consistency of the results of 10,000 permutations provides evidence for the accuracy of our research.

**TABLE 4:** The distribution of the three SNP genotypes and alleles of the *ICAM-1* gene in the cervical precancerous lesion group and the control group.

	Controls (*n* = 510)	CPL group (*n* = 684)		*P1*	P1*	*P1* ^#^	CC group (*n* = 488)		*P2*	P2*	*P2* ^#^
**rs5498**											
AA	239	318	Codominant	0.985	0.979	0.961	249	Codominant	0.320	0.162	0.127
AG	218	294	Dominant	0.995	0.982	0.989	191	Dominant	0.182	0.094	0.117
GG	48	66	Recessive	0.895	0.838	0.949	45	Recessive	0.997	0.997	0.675
A/G	0.689/0.311	0.686/0.314	Allele	0.865	0.874		0.710/0.290	Allele	0.206	0.076	
**rs3093030**											
CC	264	368	Codominant	0.744	0.693	0.906	284	Codominant	0.341	0.472	0.447
CT	205	263	Dominant	0.444	0.396	0.931	172	Dominant	0.154	0.253	0.428
TT	38	47	Recessive	0.710	0.754	0.972	29	Recessive	0.998	0.998	0.922
C/T	0.723/0.277	0.737/0.263	Allele	0.452	0.419		0.763/0.237	Allele	0.152	0.238	
**rs281432**											
CC	235	294	Codominant	0.529	0.525	0.583	222	Codominant	0.351	0.247	0.322
CG	217	286	Dominant	0.485	0.477	0.753	201	Dominant	0.355	0.343	0.629
GG	49	77	Recessive	0.294	0.286	0.644	43	Recessive	0.997	0.997	0.296
C/G	0.686/0.314	0.665/0.335	Allele	0.297	0.297		0.692/0.308	Allele	0.179	0.126	

*CPL, cervical precancerous lesions; CC, cervical cancer.*

*P1*: controls vs. cervical precancerous lesions; *P2*: cervical precancerous lesions vs. cervical cancer.

*Age was adjusted.

*^#^The *p* value was calculated using 10,000 permutations for each model to correct the multiple tests.*

*Codominant with degree of freedom 2, dominant and recessive models with degree of freedom 1.*

### Haplotype Analysis

The relationship between the haplotype distribution of the *ICAM-1* gene and cervical cancer and cervical precancerous lesions is shown in Tables 5, 6. For haplotype analyses, we found the “CTA” haplotype only in the CAC group. Furthermore, the frequency of “CTG” was significantly lower in the CAC group than in the controls (*p* = 0.007).

**TABLE 5:** Association of *ICAM-1* haplotypes with cervical cancer risk.

Haplotype	Control frequency	CC frequency	*P*	OR [95% CI]	CSCC frequency	*P1*	OR [95% CI]	CAC frequency	*P2*	OR [95% CI]
CCA	0.575	0.586	0.599	1.051 [0.873–1.266]	0.587	0.688	1.041 [0.855–1.267]	0.658	0.234	1.367 [0.815–2.293]
CTG	0.095	0.081	0.273	0.837 [0.609–1.151]	0.092	0.812	0.961 [0.693–1.333]	0.000	**0.007**	–
GCA	0.086	0.098	0.334	1.165 [0.854–1.589]	0.093	0.615	1.089 [0.781–1.519]	0.103	0.656	1.203 [0.533–2.712]
GCG	0.062	0.077	0.211	1.253 [0.880–1.784]	0.073	0.399	1.176 [0.806–1.717]	0.048	0.602	0.738 [0.235–2.324]
GTG	0.150	0.125	0.118	0.811 [0.624–1.055]	0.127	0.168	0.823 [0.623–1.086]	0.114	0.398	0.719 [0.334–1.549]
CTA	–	–	–	–	–	–		0.077	**0.0005**	5.139 [1.848–14.288]

*CC, cervical cancer; CSCC, cervical squamous cell carcinomas; CAC, cervical adenocarcinoma.*

**P*: controls vs. CC; *P1*: controls vs. CSCC; *P2*: controls vs. CAC. *p* values < 0.05 are shown in bold.*

**TABLE 6:** Association of *ICAM-1* haplotypes with cervical precancerous lesion risk.

Haplotype	Control frequency	CPL frequency	*P*	OR [95% CI]
CCA	0.575	0.547	0.172	0.888 [0.749–1.053]
CTG	0.095	0.100	0.668	1.063 [0.804–1.405]
GCA	0.086	0.109	0.062	1.308 [0.986–1.735]
GCG	0.062	0.072	0.343	1.174 [0.842–1.637]
GTG	0.150	0.139	0.459	0.915 [0.723–1.158]

*CPL, cervical precancerous lesions.*

## Discussion

Cervical cancer is a gynecological malignant tumor with high mortality (Dongol et al., 2014). Its occurrence and development are a long-term, continuous, multifactor, and multistep complex process. Persistent infection of high-risk HPV and chronic inflammation are currently recognized causes of cervical cancer (Feng et al., 2016; Yang et al., 2016; Li et al., 2017; Wang and Luo, 2018). More and more studies show that genetic variation plays an indispensable role in the pathogenesis of cervical cancer. SNPs are one of the most common types of genetic variation in humans. It is widely present in the human genome and is considered to be the third-generation genetic marker, which is closely related to the susceptibility to many diseases (Shastry, 2009; Tan, 2017). Based on the findings of previous studies, there was no research on the relationship between the SNPs of the *ICAM-1* gene and the risk of cervical cancer in the northern Chinese population. Therefore, we reported for the first time the association between *ICAM-1* gene polymorphisms and the risk of cervical cancer in the northern Chinese population.

In recent years, a large amount of evidence has shown that ICAM-1 has a dual role in the occurrence and development of tumors (Reina and Espel, 2017). ICAM-1 not only participates in the recognition and killing of tumor cells by immune cells but also plays a certain role in the process of tumor evading the body’s immunity. ICAM-1 can not only mediate the nonspecific adhesion between tumor cells and T cells and facilitate the recognition of tumor cells by T cells but also bind to the LFA-1 ligand on the surface of T cells to form a complex ICAM-1/LFA-1. Tumor necrosis factor-α (TNF-α) and interleukin-1 (IL-1) secreted by ICAM-1/LFA-1 can not only kill tumor cells directly but also significantly upregulate the expression of ICAM-1 on the surface of tumor cells, to promote the recognition of immune cells to tumor cells and further expand its killing effect (Nakayama et al., 2011). Therefore, some scholars believe that the formation of the ICAM-1/LFA-1 complex is a positive feedback mechanism in the immune process of tumor cells, which helps to eliminate tumor cells in the body (Ren et al., 2010). However, sICAM-1, as a competitive inhibitor of ICAM-1, can competitively bind to the LFA-1 ligand, inhibit the formation of the ICAM-1/LFA-1 complex, weaken its killing effect, and cause tumor cells to evade the body’s immune surveillance (Dustin et al., 1986; Nasu et al., 1997; Li and Gao, 2008), resulting in tumor development and metastasis.

This study focused on the relationship between the rs5498, rs3093030, and rs281432 polymorphisms of the *ICAM-1* gene and the susceptibility of cervical cancer in the northern Chinese population. Our results indicated that rs3093030 was significantly associated with the susceptibility of cervical cancer, that is, carrying the T allele and the dominant model has a protective effect on the susceptibility of cervical cancer (*p* = 0.042, OR = 0.811, 95% CI: 0.663∼0.993; *p* = 0.040, OR = 0.769, 95% CI: 0.598–0.988). It was speculated that the protective effect may be related to the increase of sICAM-1 concentration by rs3093030 (Bielinski et al., 2011; Lin et al., 2013; Ghasemian et al., 2015). The rs3093030 located at the 3′ end of the *ICMA-1* gene has no known function (Bielinski et al., 2008). Therefore, rs3093030 may affect the concentration level of sICAM-1 through a special mechanism, thereby affecting the competitive effect with ICAM-1. On the one hand, ICAM-1 can bind more to the ligand LAF-1 and exert its killing effect in tumor cells. On the other hand, it also reduces the immune evasion of tumor cells, inhibits the occurrence and development of tumors, and protects the susceptibility of tumors. However, after Bonferroni correction, we did not find a significant association between the rs3093030 polymorphisms and cervical cancer. Therefore, it is necessary to further study whether there is an association between rs3093030 and the susceptibility to cervical cancer. In addition, our study did not find that rs5498 was associated with the risk of cervical cancer, but subgroup analysis showed that rs5498 was associated with the risk of CAC. In the codominant model and dominant model, it has a protective effect on the susceptibility of CAC (codominant *p* = 0.028; dominant *p* = 0.011, OR = 0.374, 95% CI: 0.175–0.799), and its mutant gene G can also be used as a protective factor to reduce the risk of CAC (*p* = 0.009, OR = 0.045, 95% CI: 0.221–0.827). The logistic regression analysis showed that rs5498 was significantly associated with CAC (codominant *p* = 0.041; dominant *p* = 0.012, OR = 0.362, 95% CI: 0.164–0.799; allele *p* = 0.016, OR = 0.431, 95% CI: 0.218–0.853). Meanwhile, the correlation remained after 10,000 permutation tests and after Bonferroni correction. rs5498 may be closely related to the pathological classification of cervical cancer. Rs5498 was located 3 bp upstream of the splice donor site, Bielinski et al. (2011) confirmed that when this site was mutated, it will cause the destruction of the splice site, causing the G mutant gene vector to produce a higher concentration of sICAM-1. This process will help tumor cells to escape from the body’s immune killing and immune escape occurs. However, in our study, false-positive results due to the small sample size of the CAC group were not ruled out. Therefore, the impact of rs5498 on cervical cancer was controversial, and further classification and repeated studies with large sample sizes were needed. Bielinski et al. confirmed that rs281432 was associated with sICAM-1 (Bielinski et al., 2011). In addition, it has been reported that elevated serum sICAM-1 levels may contribute to the diagnosis of malignancy in suspected breast neoplastic diseases (Altomonte et al., 1999). However, no significant association between rs281432 SNP and cervical cancer was found in this study, or in the subgroup.

This study also analyzed the relationship between the SNPs of the *ICAM-1* gene and cervical precancerous lesions in the Han population in northern China. The results showed that the rs5498, rs3093030, and rs281432 of the *ICAM-1* gene were not significantly associated with the susceptibility of cervical precancerous lesions. It was inconsistent with the research results reported by Sun et al. (2016) that rs281432 and rs5498 can increase the risk of cervical precancerous lesions. The reasons for the two different research results may be attributed to the following points. Firstly, limited sample sizes, living environment (such as lifestyle, education methods, and socioeconomic factors), and ethnic difference may change the genetic effects of *ICAM-1* gene mutations. Secondly, other genes or factors may be involved in the development of cervical cancer. Last but not least, the relationship between the *ICAM-1* gene and the risk of cervical cancer may not be achieved by affecting the structure and function of encoding protein.

To assess the combined effect of SNPs on the risk of cervical cancer and to identify the possible risk haplotypes in the population, we performed multilocus haplotype analysis. The haplotype analysis showed that “CTG” was significantly associated with CAC risk (*p* = 0.007). The result of the above haplotype frequency distribution was significantly lower in the CAC cases than in the controls. The haplotype “CTG” was found to significantly reduce the risk of CAC.

Although Sun et al. (2016) confirmed that *ICAM-1* gene polymorphism is significantly associated with cervical cancer, the sample size is relatively small, including only 91 cases of cervical cancer, 63 cases of cervical precancerous lesions, and 290 healthy controls. A large sample size is very important in exploring and verifying the potential mechanism of cervical cancer in Chinese women. This study analyzes and elaborates from multiple angles such as cervical cancer subgroups and cervical precancerous lesions, which will play an important role in future research on cervical cancer with different pathological types. This study confirmed for the first time the correlation between *ICAM-1* gene polymorphisms and cervical cancer in the northern Chinese Han population. This discovery can guide us to develop new understanding and insights into the mechanism and pathological process of cervical cancer. In addition, this study also has some unavoidable limitations. First of all, this study uses a case–control study, and selection bias cannot be avoided. Second, the sample size of the CAC group in this study is small, so it is necessary to conduct a larger sample size replication study to confirm the results. Finally, our study population is limited to Han women in northern China. In order to improve this study, people from different regions and ethnic backgrounds should be included.

## Data Availability Statement

The datasets presented in this study can be found in online repositories. The names of the repository/repositories and accession number(s) are: FigShare, doi:106084/m9.figshare.14039183.

## Ethics Statement

Written informed consent was obtained from the individual(s), and minor(s)’ legal guardian/next of kin, for the publication of any potentially identifiable images or data included in this article.

## Author Contributions

YF: data collection, data analysis, and manuscript writing. XL and QM: data analysis. SZ, MZ, SL, and LF: data collection. JT and LS: project development. All authors contributed to the article and approved the submitted version.

## Conflict of Interest

The authors declare that the research was conducted in the absence of any commercial or financial relationships that could be construed as a potential conflict of interest.

## Publisher’s Note

All claims expressed in this article are solely those of the authors and do not necessarily represent those of their affiliated organizations, or those of the publisher, the editors and the reviewers. Any product that may be evaluated in this article, or claim that may be made by its manufacturer, is not guaranteed or endorsed by the publisher.

## Footnotes

1
http://www.biowing.com.cn

2
http://frodo.wi.mit.edu/

3
http://analysis.bio-x.cn/myAnalysis.php

4
http://www.Cog-genomics.org/plink/1.9/
